# Genotyping-by-sequencing approach indicates geographic distance as the main factor affecting genetic structure and gene flow in Brazilian populations of *Grapholita molesta* (Lepidoptera, Tortricidae)

**DOI:** 10.1111/eva.12257

**Published:** 2015-04-13

**Authors:** Karina Lucas Silva-Brandão, Oscar Arnaldo Batista Neto e Silva, Marcelo Mendes Brandão, Celso Omoto, Felix A H Sperling

**Affiliations:** 1 Laboratório de Melhoramento de Plantas, Centro de Energia Nuclear na Agricultura, Universidade de São PauloPiracicaba, SP, Brazil; 2 Departamento de Entomologia e Acarologia, Escola Superior de Agricultura ‘Luiz de Queiroz’, Universidade de São PauloPiracicaba, SP, Brazil; 3 Centro de Biologia Molecular e Engenharia Genética, Universidade Estadual de CampinasCampinas, SP, Brazil; 4 Department of Biological Sciences, CW405 Biological Sciences Centre, University of AlbertaEdmonton, AB, Canada

**Keywords:** genetic structure, host plant association, oriental fruit moth, single-nucleotide polymorphisms

## Abstract

The oriental fruit moth *Grapholita molesta* is one of the major pests of stone and pome fruit species in Brazil. Here, we applied 1226 SNPs obtained by genotyping-by-sequencing to test whether host species associations or other factors such as geographic distance structured populations of this pest. Populations from the main areas of occurrence of *G. molesta* were sampled principally from peach and apple orchards. Three main clusters were recovered by neighbor-joining analysis, all defined by geographic proximity between sampling localities. Overall genetic structure inferred by a nonhierarchical amova resulted in a significant Φ_ST_ value = 0.19109. Here, we demonstrate for the first time that SNPs gathered by genotyping-by-sequencing can be used to infer genetic structure of a pest insect in Brazil; moreover, our results indicate that those markers are very informative even over a restricted geographic scale. We also demonstrate that host plant association has little effect on genetic structure among Brazilian populations of *G. molesta*; on the other hand, reduced gene flow promoted by geographic isolation has a stronger impact on population differentiation.

## Introduction

Interactions among herbivorous insects and their host plants define most of the dynamics of phytophagous insect populations, which correspond to ca. of 43% of recognized insect species (Grimaldi and Engel 2005). For example, the ability to feed on plants has influenced processes of diversification and speciation in insects (Mitter et al. 1988), due in part to barriers to gene flow among individuals feeding on different hosts (Funk 1998; Dres and Mallet 2002). Populations of polyphagous insects that feed on distinctive host plants may become genetically isolated (Martel et al. 2003; Machado et al. 2008), in a process leading to increased reproductive isolation (Dres and Mallet 2002). If the genetic differentiation is adaptive, populations from different host plants will differ at key genes, and the challenge is to identify the specific genes involved in differentiation and speciation (Beaumont and Balding 2004).

Populations of pest insects associated with different hosts have been characterized for several species (Pashley 1986; Shufran et al. 2000; Perring 2001; Nagoshi et al. 2007), and genetic divergence related to host plant use, which can be considered a case of ecological speciation (Matsubayashi et al. 2010), has been investigated in some Lepidoptera (Emelianov et al. 1995; Groman and Pellmyr 2000; Martel et al. 2003; Machado et al. 2008). One prediction of ‘ecological speciation’ models is that pairs of populations feeding on distinctive host plants will be more genetically diverse than pairs feeding on the same host (Funk 1998). Host races (as defined by Dres and Mallet (2002)) of the apple maggot fly *Rhagoletis pomonella* (Walsh) (Diptera, Tephritidae) are a classic example of this prediction (Bush 1969; Feder et al. 1988; Mcpheron et al. 1988). Host races (or ‘host forms’) have also been suggested for the noctuid moth *Spodoptera frugiperda* (J. E. Smith), based on ecological, genetic and physiological differences (Pashley 1993; Busato et al. 2004; Nagoshi et al. 2007; Juarez et al. 2014), and for the tortricid moth *Cydia pomonella* (L.), based on divergent biological responses and oviposition behavior adaptations related to larval host plants (Phillips and Barnes 1975; Barnes 1991).

The oriental fruit moth *Grapholita molesta* (Busck) (Lepidoptera: Tortricidae) is, together with *C. pomonella*, one of the major pests in Brazil of stone and pome fruit species, particularly those in the Rosaceae (apple, peach, pear, nectarines) (Salles 2001; Silva et al. 2010). The putative indigenous area of *G. molesta* includes China (Kirk et al. 2013; Zheng et al. 2013), although its native host plants have a broader distribution throughout all Central Asia (Rothschild and Vickers 1991). From China, *G. molesta* broadened its distribution, and currently, it is found across temperate regions of Asia, Europe, Americas, Africa, and Australia (Rothschild and Vickers 1991; Kirk et al. 2013). In South America, the oriental fruit moth was recorded simultaneously in Argentina and Brazil, in the state of Rio Grande do Sul, around 1929 (Rothschild and Vickers 1991); after which it extended its range to Uruguay and Chile (Salles 2001). In Brazil, it is currently found throughout the South Central region (Salles 2001).

There is sparse evidence of lineages associated with host plant preference in *G. molesta* (Rothschild and Vickers 1991); however, populations from the eastern United States of America show oviposition preference for peach plants independently of previous host (Myers et al. 2006), and larval development is faster in both fruits and growing terminal shoots of peach than in the same parts of apples (Myers et al. 2007). Peach and apple volatiles also seem to attract females of *G. molesta* differently (Piñero and Dorn 2009).

*Grapholita molesta* in its assumed native range in China has shown differences in genetic structure between populations collected from peach and those collected from apple and pear in the late season (Zheng et al. 2013). However, a broader study found no association between genetic structure and the host species that were used by sampled populations (Kirk et al. 2013). Additional population genetic studies did not focus on host plant differences and have found low to moderate overall genetic structure for both South African (Timm et al. 2008) and Italian (Torriani et al. 2010) populations. All these studies rely on anthropogenic movement of fruits, bins, and nursery material to explain the displacement of individuals of *G. molesta*, as the species has low dispersal capability but retains the ability to disperse among orchards (Hughes and Dorn 2002).

The main objective of this study was to characterize the genetic variability of populations of *G. molesta* sampled from different hosts (apple, peach and nectarine) in the main regions of occurrence of this species in Brazil, applying for the first time the genotyping-by-sequencing approach of simultaneous discovery of single-nucleotide polymorphisms (SNPs) and individual genotyping (Elshire et al. 2011) to estimate genetic variation and structure of a pest insect species in Brazil. With these markers, we test whether populations are genetically structured by host species associations (peach and apple) or other factors such as geographic distance.

## Material and methods

### Sampling

**Figure 1:**
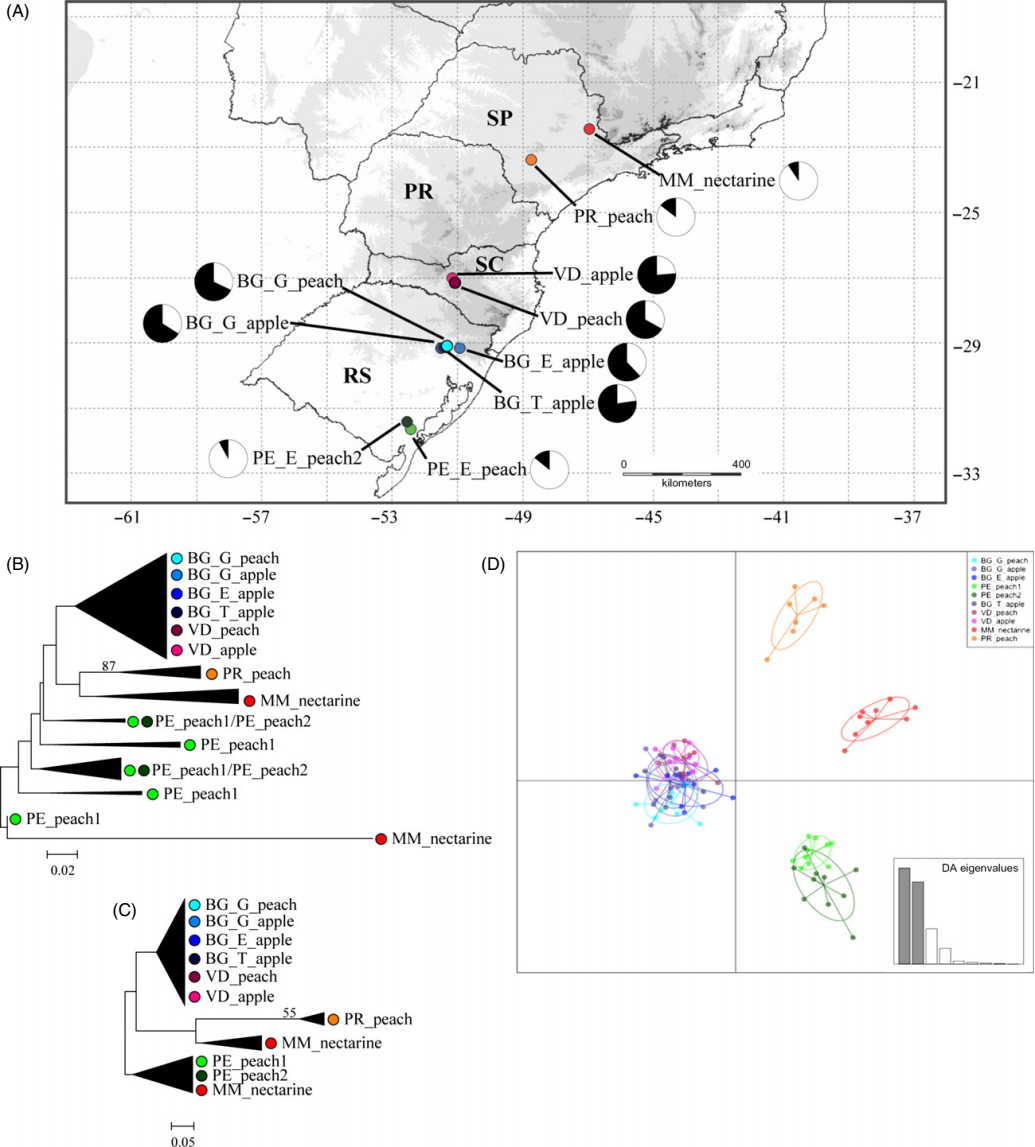
Sampling localities of *Grapholita molesta* in Brazil, with the frequency of individuals belonging to clusters 1 (in white) and 2 (in black) for each locality, as recovered in the Bayesian assignment test implemented in Structure (A); neighbor-joining topologies based on (B) all 1226 loci, and (C) 211 outliers; DAPC of sampling localities (D).

A total of 96 individuals of *G. molesta* from 10 localities, separated by 1.5–1140 km, were sampled between December 2011 and April 2012, mainly from peach and apple orchards throughout the main fruit producer states in Brazil (Fig.1A, Table1). Male adults were sampled in Delta traps with synthetic sex pheromone (Isca Tecnologias Ltda., Ijuí, RS, Brazil), located 1.70 m above ground (Hickel et al. 2003). Captured individuals were removed daily from traps for 1 week and immediately immersed in 100% ethanol. Samples were kept at −20°C until DNA extraction.

**Table 1:** Sample data: locality, host plant, locality code, coordinates, collection date, and number of individuals sampled per locality.

Locality	Host plant	Code	Latitude/Longitude	Date	*n*
Bento Gonçalves, RS	Peach	BG_G_peach	29°7′S/51°24′W	Jan/2012	10
Bento Gonçalves, RS	Apple	BG_G_apple	29°8′S/50°55′W	Feb/2012	10
Bento Gonçalves, RS	Apple	BG_E_apple	29°10′S/51°31′W	Feb/2012	10
Pelotas, RS	Peach	PE_peach1	31°40′S/52°25′W	Feb/2012	10
Pelotas, RS	Peach	PE_peach2	31°25′S/52°32′W	Feb/2012	10
Bento Gonçalves, RS	Apple	BG_T_apple	29°7′S/51°25′W	Feb/2012	10
Videira, SC	Peach	VD_peach	27°0′S/51°9′W	Jan/2012	9
Videira, SC	Apple	VD_apple	27°0′S/51°9′W	Apr/2012	9
Mogi Mirim, SP	Nectarine	MM_nectarine	22°25′S/46°57′W	Apr/2012	9
Paranapanema, SP	Peach	PR_peach	23°23′S/48°43′W	Dec/2011	9
Total					96

### DNA extraction and genotyping-by-sequencing protocol

Total genomic DNA was extracted from whole insects using the standard procedure of the DNeasy Blood and Tissue Kit (Qiagen AG, Hombrechtikon, Switzerland). DNA was eluted in 100 μL AE buffer and stored at −20°C. Final concentrations and 280/260 and 260/230 ratios were estimated with a NanoDrop UV spectrophotometer (Techno Scientific, Wilmington, DE, USA). DNA amount per sample was normalized to 20 ng/μL.

Genotyping-by-sequencing libraries were constructed using standard protocols (Elshire et al. 2011; Poland et al. 2012), with minor modifications, in the Institut de Biologie Intégrative et des Systèmes (IBIS), in University of Laval (Quebec city, Canada). DNA was digested with both high-fidelity PstI (New England Biolabs, Whitby, ON, Canada) and MspI (New England Biolabs) restriction enzymes. Ninety-six bar-coded P1 adapters were ligated on the PstI cut site for each individual sample. A common adapter (adapter 2) was ligated onto the MspI cut site of all samples. Two sets of 48 samples were pooled for multiplexed PCRs, using standard forward primer A and modified reverse primer C with 1 nt for complexity reduction (Sonah et al. 2013). PCR products were purified with Agencourt Ampure XP beads (Beckman-Coulter, Inc., Brea, CA, USA). DNA amount was estimated with a NanoDrop UV spectrophotometer, normalized with duplex-specific nuclease (Shagina et al. 2011), and re-amplified with standard primers A and C. These second PCR products were purified and DNA amount estimated as above. The two libraries with 48 samples each were sequenced in two lanes of an Illumina HiSeq2000 (Illumina, Inc., San Diego, CA, USA) using 100-bp single-end reads, at the McGill University and Génome Québec Innovation Centre (Montreal, Canada).

### SNP calling pipeline and quality filtering

We used the TASSEL 3.0 UNEAK pipeline for organisms without a reference genome for SNP calling (Lu et al. 2012, 2013). The pipeline procedure aligns reads by barcode type, trims barcodes off to give sequences of 64 bp, merges tag files of the same individual (default minimum number of times a tag must be present to be output = 5), gives pairwise alignment via the network filter (ETR = 0.03), and assigns genotypes to each individual (allele frequency minimum = 0.05; maximum = 0.5). In the end, the pipeline generated a HapMap genotype with single letters (hmp) for each individual. Two other output files were generated, the first with tag counts of the SNPs in each individual (hmc) and the other with sequences of the SNP tags (fas).

Data in the hmp file were used to compute the minimum number of reads per individual (x*) needed to ensure that the probability (*α*) of misclassifying heterozygotes as homozygotes was ≤0.05 (Chenuil 2012). Accordingly:

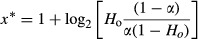


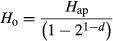


where *H*_o_ = observed heterozygosity; *H*_ap_ = ‘apparent heterozygosity’ or proportion of heterozygotes per locus; *d *= observed sequence depth.

Only loci with sequence depth above the estimated x* value were retained for posterior analyses.

Additional filtering included removing loci absent in ≥5% of individuals and individuals with ≥10% of missing loci. The program Genepop v. 4. 2 (Raymond and Rousset 1995; Rousset 2008) was used to infer which loci were under Hardy–Weinberg disequilibrium for all sampled localities (*P *<* *0.05). Loci under disequilibrium were pruned from the data matrix. Posterior analyses were carried out using loci and individuals that conformed to the above-mentioned parameters. File conversions, to allow the use of various population genetics software, were accomplished using PGDSpider v. 2.0.5.1 (Lischer and Excoffier 2012).

### Outlier analyses

The program Lositan (Antao et al. 2008) was used to detect loci under selection based on the neutral distribution of *F*_ST_ values for all loci in relation to *H*_e_ (expected heterozygosity). Any locus with *F*_ST_ higher or lower than the neutral distribution (outlier) is considered a candidate for being under selective pressure (Beaumont and Nichols 1996). Lositan was first run using all loci under attempted neutral mean *F*_ST_, 50 000 simulations, 99% confidence interval, infinite alleles mutation model, and false discovery rate of 0.1%, following the procedure described in Antao et al. (2008), to lower the bias on the estimation of the mean neutral *F*_ST_ by eliminating extreme loci from the estimation. After the first run, all loci that were outside the confidence interval were removed, and the mean neutral *F*_ST_ was recalculated. Only the supposed neutral loci were used in this run under the same parameters as above. The third run used all loci and the newly calculated neutral *F*_ST_, with all other parameters maintained. Loci recovered as outliers in the last run were inferred to be under selection.

A Bayesian approach was also applied to identify loci under selection using the program BayeScan v. 2.1 (Foll and Gaggiotti 2008). We ran three analyses under default parameters, and loci were considered to be under selection if they were found in all three analyses with *q* value < 0.05.

### Population genetics analyses

The program MEGA v. 5.0 (Tamura et al. 2011) was used to estimate the genetic distance among *G. molesta* individuals based on (i) all loci and (ii) loci identified as being under selection using the program Lositan. This approach was applied to compare the power of discrimination of individuals within populations of likely non-neutral (or adaptive) markers and putatively neutral markers (Kirk and Freeland 2011; Keller et al. 2012). MEGA was used to estimate the best evolutionary model explaining the two datasets, and to infer a distance tree using the neighbor-joining (NJ) algorithm (Saitou and Nei 1987). Branch supports were inferred with 1000 bootstrap replicates.

A discriminant analysis of principal components (DAPC, Jombart et al. 2010) was applied to provide a visual evaluation of the genetic structure of Brazilian populations of *G. molesta*, using the R package adegenet (Jombart 2008). Sampling localities were used as prior groups, and all loci were used as input.

Overall genetic structure was estimated by a nonhierarchical analysis of molecular variance (amova) using the software Arlequin v. 3.5 (Excoffier and Lischer 2010). Hierarchical amova was conducted among the following: (i) clusters of sampling localities found using DAPC and NJ and (ii) host plants from which samples were collected (apples versus peach; samples from nectarine were excluded from the analysis as they were from only one locality). Genetic structure was interpreted from the Φ statistics associated with different hierarchical levels in which variation is distributed (Excoffier et al. 1992). Significance of the Φ_ST_ values was evaluated using the following parameters: 10 000 permutations, computed distance matrix using pairwise difference, and gamma *a* value = 0. Slatkin (Slatkin 1995) pairwise *F*_ST_ values were also estimated in Arlequin. The same program was used to run a Mantel test (Mantel 1967), with 10 000 permutations, to estimate the correlation of pairwise linearized distances with a matrix of linear geographic distances to test the hypothesis of genetic isolation by geographic distance (isolation by distance, IBD).

Genetic structure was also estimated using the Bayesian assignment test implemented in the program Structure v. 2.3.3 (Pritchard et al. 2000). The number of clusters (*K*) was estimated with putatively neutral loci. Each nucleotide was numerically coded as follows: A = 1, T = 2, C = 3, G = 4, all other characters = 0, and missing data = −9. Run parameters included 25 runs with 500 000 iterations following a burn-in period of 50 000 iterations for *K* = 1–12, under the ‘admixture ancestry model’ and allele frequencies ‘correlated’. The Δ*K* of Evanno (Evanno et al. 2005) was calculated using the application Structure Harvester v. 0.6.94 (Earl and Vonholdt 2012) to estimate the number of clusters (*K*). The frequency of individuals in each cluster was visualized using the programs CLUMPP v. 1.1.2 (Jakobsson and Rosenberg 2007) and Distruct v. 1.1 (Rosenberg 2004).

## Results and discussion

The SNP calling pipeline recovered 23 765 SNPs. After all filtering procedures and Hardy–Weinberg equilibrium tests, 1226 SNPs were maintained in our matrix, for 93 individuals. Subsequent analyses were carried out with that matrix.

**Figure 2:**
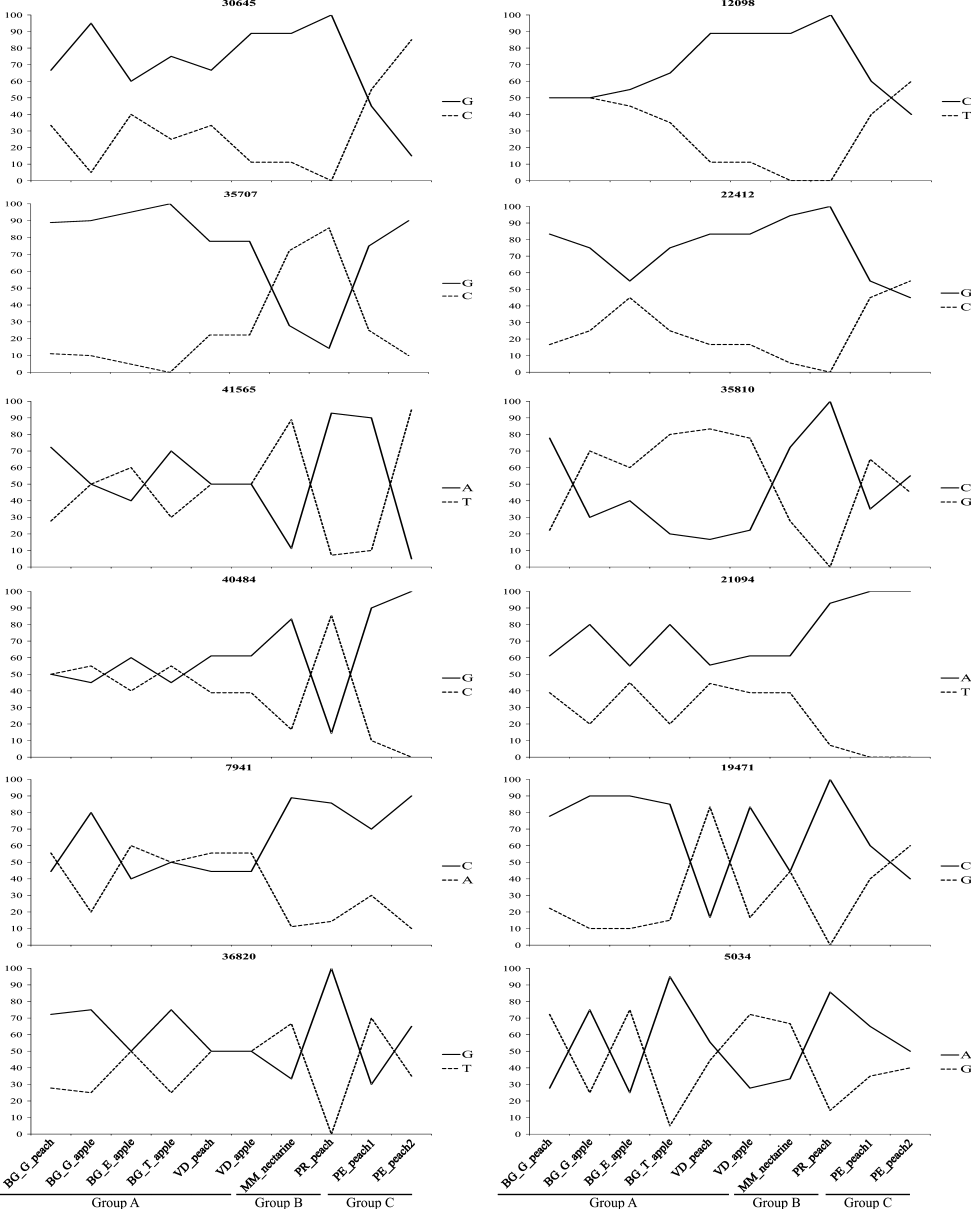
Frequency of polymorphisms in 12 loci putatively under selection in populations of *Grapholita molesta*.

Lositan recovered 211 loci that were putatively under selection (outliers). BayeScan recovered 12 outliers, all of them with positive values of *α*, which is indicative of diversifying selection, and *P *>* *0.85, indicative of ‘substantial’ to ‘decisive’ evidence of selection. All 12 loci were also recovered by Lositan, with *P *>* *0.99, which the program suggests as candidates for positive selection loci. Frequency of nucleotide polymorphisms of each of the 12 loci varied strongly among populations (Fig.2).

The best model fit explaining the data matrix composed of all loci was K2 + G+I (Kimura-2-parameters + gamma parameter + invariable sites). For loci inferred as being under selection with the program Lositan, the best model was K2 + I. Three main clusters were recovered when those outliers were used to construct the neighbor-joining (NJ) topology (Fig.1C): group A is composed of the four samples from Bento Gonçalves, RS + the two samples from Videira, SC; group B is composed of the two samples from São Paulo state; and group C is composed of the two samples from Pelotas, RS. In general, clusters were defined by geographic proximity between sampling localities. Clusters were less resolved in the topology obtained with all loci (Fig.1B); indeed, non-neutral markers alone assigned individuals to their original population better than all markers combined. These findings agree with the growing discussion on the advantages of using data from non-neutral molecular markers in studies of molecular ecology and for population structure inferences (Kirk and Freeland 2011).

DAPC recovered the same clusters achieved by the NJ topology based only on outliers, with the two samples from São Paulo state more distant than other samples within their respective clusters (Fig.1D). For both DAPC and NJ analyses, host plants from which samples were collected did not delimit groups. Instead, clusters followed a geographic pattern (Fig.1). That arrangement was also indicated by a significant pattern of isolation by distance (*P *=* *0.0081), as the correlation between geographic distances and Slatkin's pairwise linearized distances inferred by the Mantel test explains most of the variation (*r*^2^ = 0.5828).

Overall genetic structure inferred by a nonhierarchical amova resulted in a significant Φ_ST_ value = 0.19109 (*P *<* *0.001). The hierarchical amova considering host association resulted in a Φ_ST_ = 0.20359 (*P *<* *0.001); however, only 5% of the variation was due to host plants, which indicates a small role of hosts in shaping population structure; 15% of the variation arose among populations within groups, and the remaining 80% arose within populations. The hierarchical amova among the three clusters found with DAPC and NJ analyses resulted in a Φ_ST_ = 0.25121 (*P *<* *0.001), which is considered a high level of genetic structure; 19% of the variation is among groups, while 75% is within populations.

The Bayesian assignment test conducted in Structure recovered Δ*K* = 2 with the highest likelihood, which indicates two genetic clusters (Fig.1A). The frequency of individuals in each cluster in the sampled localities agreed with the pattern of grouping found with NJ analysis, that is, most individuals in group A belong to cluster 1 (in white), while most individuals in groups B and C belong to cluster 2 (in black) (Fig.1A). This result suggests a geographic basis for genetic structure in Brazilian populations of *G. molesta*.

The genotyping-by-sequencing (GBS) technique (Elshire et al. 2011; Poland et al. 2012) has revolutionized the field of population genomics by the huge amount of genetic information that can be easily gathered for the genome of any organism of interest, at a relatively low cost (Davey et al. 2011). With the high number of markers found by GBS, it is possible to estimate genetic variation and structure even at a relatively restricted geographic scale (Keller et al. 2012). For this reason, it is imprudent to compare genetic structure statistical values obtained with GBS markers to those obtained with relatively less informative markers. The high value of Φ_ST_ that we found for Brazilian populations of *G. molesta* (0.19109) is close to the value found at a continental scale (*F*_ST_ = 0.219) based on microsatellites (Kirk et al. 2013), although it is lower than the similar metric *G*_ST_ found for South African populations using AFLPs (0.279, Timm et al. 2008). For Italian populations, microsatellites revealed an *F*_ST_ = 0.042 at a restricted geographic scale (Torriani et al. 2010). At this point, there are still few studies with insects using SNPs obtained by next-generation sequencing for further comparisons. One instance is the *F*_ST_ found for 28 populations of the Phasmatoidea stick insect *Timema cristinae*, based on 86 130 SNPs, which was 0.111 (*P *=* *0.001) (Nosil et al. 2012).

Slatkin's (1995) linearized pairwise *F*_ST_ values ranged from 0 to 0.62944 among all samples. The highest significant value was found between samples collected in Bento Gonçalves (BG_G_peach) and in Paranapanema, SP (PR_peach), approximately 688 km apart (Table2). Average Slatkin's pairwise *F*_ST_ among peach samples was 0.293 (SD = 0.187), among apple samples was 0.213 (SD = 0.198), and among peach versus apple samples was 0.223 (SD = 0.192). Similar pairwise *F*_ST_ values among pairs of samples, from the same or different hosts, are in accordance with a weak effect of host plants in the genetic differentiation among populations.

**Table 2:** Slatkin pairwise *F*_ST_ values among all sampling localities of Brazilian populations of *Grapholita molesta*.

Code	1	2	3	4	5	6	7	8	9
1. BG_G_peach	–								
2. BG_G_apple	0.02729	–							
3. BG_E_apple	0.01139	0.01432	–						
4. PE_peach1	**0.13020**	**0.09577**	0.03546	–					
5. PE_peach2	**0.11002**	**0.09034**	0.03168	0.01049	–				
6. BG_T_apple	0	0.01288	0.00588	**0.11749**	**0.07106**	–			
7. VD_peach	**0.36763**	**0.33308**	**0.22580**	**0.20574**	**0.24929**	**0.24585**	–		
8. VD_apple	**0.45445**	**0.45218**	**0.32408**	**0.35678**	**0.38433**	**0.38639**	**0.04238**	–	
9. MM_nectarine	**0.31810**	**0.27498**	**0.26590**	**0.23189**	**0.29380**	**0.34098**	**0.13543**	**0.24902**	–
10. PR_peach	**0.62944**	**0.54343**	**0.46814**	**0.39783**	**0.45292**	**0.50505**	**0.37909**	**0.42331**	**0.24498**

Bold numbers are significant values under *α* = 0.05.

Host plant association does not affect genetic structure between Brazilian populations of *G. molesta*, as found for other populations (Kirk et al. 2013). Instead, geographic isolation has a stronger function in population differentiation. Even at a broader geographic range, Kirk et al. (2013) found that two geographically separated Brazilian populations form two different genetic clusters with European populations.

Geographic isolation is known to reduce gene flow among populations of phytophagous insects and is a factor usually associated with low dispersal capability (Peterson and Denno 1998), as is the case for *G. molesta* (Hughes and Dorn 2002). Long-distance dispersal is unlikely for this species, and close relatives should be constrained within their neighbor orchards, sequentially using all suitable host plants available at the time. On the other hand, early long-distance anthropogenic dispersal might be responsible for the initial spread of these insects in Brazilian orchards. That may be the case for populations from Santa Catarina state (SC) that grouped with samples from Rio Grande do Sul (RS). There was no record of *G. molesta* in Santa Catarina until 1982, and since then moths have been trapped annually in the region, and it is now considered an important pest in apple orchards, damaging up to 90% of fruits (Reis et al. 1988). It is likely that those samples were originally from the neighbor state, and the present genetic similarity between those populations is due to historical dispersal instead of current gene flow.

This is the first time that SNPs gathered by the genotyping-by-sequencing technique have been applied to infer genetic structure of a pest insect in Brazil. The results we found so far indicate that these markers are very informative even at a restricted geographic scale. Furthermore, the main advantage in using this technique is the opportunity to infer loci under selection, and to test the potential of putatively non-neutral markers to differentiate populations. A further advantage is the promising possibility for annotation and linking of inferred non-neutral markers to important biological functions or biochemical processes, especially if a reference genome is available. Such identification, however, is limited by the availability of reliable annotated genomes, which has improved in recent years due to the popularization of next-generation sequencing, making annotation of outliers loci more feasible in the near future.

## Data archiving statement

Raw sequencing reads were deposited in the BioProject, accession number: PRJNA277038, ID: 277038, and BioSample, under the following accession numbers: SAMN03386922–SAMN03386931.
